# Switching Hemispheres: A New Migration Strategy for the Disjunct Argentinean Breeding Population of Barn Swallow (*Hirundo rustica*)

**DOI:** 10.1371/journal.pone.0055654

**Published:** 2013-01-31

**Authors:** Belen Garcia-Perez, Keith A. Hobson, Rebecca L. Powell, Christopher J. Still, Gernot H. Huber

**Affiliations:** 1 Department of Biology, University of Saskatchewan, Saskatoon, Saskatchewan, Canada; 2 Environment Canada, Saskatoon, Saskatchewan, Canada; 3 Center for Energy, Environment and Sustainability, Wake Forest University, Winston-Salem, North Carolina, United States of America; 4 Forest Ecosystems and Society of Geography, Oregon State University, Corvallis, Oregon, United States of America; 5 Department of Ecology and Evolutionary Biology, Cornell University, Ithaca, New York, United States of America; University of Osnabrueck, Germany

## Abstract

**Background:**

Barn Swallows (*Hirundo rustica*) breed almost exclusively in the Northern Hemisphere. However, since the early 1980's, a small disjunct breeding population has become established in eastern Argentina, presumably by birds previously derived from those breeding in North America. Currently, it is unknown where these individuals go following breeding and how they have adjusted to a reversal in phenology. Their austral wintering period corresponds to the breeding period of the northern ancestral population and so they can potentially return to these more traditional breeding sites or they may occupy other South American wintering regions left vacant by conspecifics returning to the Northern Hemisphere.

**Principal Findings:**

We used a three-isotope (*δ* ^13^C, *δ* ^15^N, *δ* ^2^H) approach to investigate potential wintering areas in Central and South America of individuals breeding in Argentina. Feather isotope values differed from those expected and measured at local breeding sites in Argentina indicating molt after the austral breeding period and away from the breeding grounds. Potential molting origins were identified applying likelihood-based assignment methods to a *δ* ^2^H isoscape for South America and dichotomous prior information on the distribution of C3 and C4 vegetation types based on modeled vegetation-*δ* ^13^C values. Barn Swallows now breeding in Argentina have changed their migratory behavior but presumably use the same cues as those used by the ancestral population, molting their feathers during the austral winter, likely in north-eastern South America.

## Introduction

While avian migration systems appear well established, involving the regular periodic movement by individuals of several thousand kilometers [1], there are several intriguing cases whereby individuals and populations respond rapidly to changing environmental circumstances leading to a modification of their migratory strategy (e.g. [2], [3], [4]). Such responses presumably confer significant benefits to those founder populations thereby promoting an evolutionary change in migratory behavior [5]. Additionally, the appearance of new migratory traits usually involve further physiological and behavioral changes such as timing of reproduction and onset of molt, associated with changes in circannual rhythms as an adaptation to new climates, photoperiod or other circumstances [6]–[8].

However, there are generally few opportunities to examine the evolution of such adaptive processes due to the rarity of well-documented cases of new migratory systems in nature. Moreover, until recently, it has been extremely difficult to infer movements of small birds at continental scales due to limitations in conventional approaches to tracking animal movements [9]. Here, we present the results of a study of a recently established disjunct breeding population of Barn Swallow (*Hirundo rustica*) in Argentina [10] whose movements were inferred using a multiple stable isotopic forensic examination of their feathers. The North American (i.e. ancestral) breeding population undergoes a complete annual molt of flight feathers during the non-breeding season in South America [11]. Recent genetic studies indicate that this new population was initially derived from the North American breeding population through colonization by migratory individuals [12] but nothing is known of how new migratory and life history strategies may have since rapidly evolved.

## Methods

### Ethics statement

All animals in this study were handled in accordance with The North American Bird Banding Council guidelines. This work was conducted under all necessary permits for capture and sample collection granted by the correspondent Argentinean wildlife authority in Buenos Aires province (Dirección de Administración de Áreas Protegidas y Conservación de la Biodiversidad).

### Samples

We sampled 100 breeding Barn Swallows during the austral summer (November-January) of 2006 and 2007 at colonies along the Atlantic coast of Buenos Aires province, Argentina. Tail feathers clearly grown prior to breeding were identified based on wear [11] and collected for all captured birds. For some individuals captured at the beginning of the breeding season tail feathers were induced to regrow and were then collected later in the season. Other than these induced new feathers, no molt was observed during the (austral) breeding season.

### Stable isotope measurements

All feathers were cleaned of surface oils in 2∶1 (v/v) chloroform:methanol solvent rinse and prepared for *δ* ^2^H, *δ* ^13^C and *δ* ^15^N analysis at the Stable Isotope Laboratory of Environment Canada, Saskatoon, Canada. The non- exchangeable hydrogen of feathers was determined using the method described by [13] and using two calibrated keratin hydrogen-isotope reference materials. Hydrogen isotopic measurements were performed on H_2_ gas derived from high-temperature (1350°C) flash pyrolysis of 350±10 µg feather subsamples and keratin standards using continuous-flow isotope-ratio mass spectrometry. Measurement of the two keratin laboratory reference materials (CBS, KHS) corrected for linear instrumental drift were both accurate and precise with typical mean *δ* ^2^H ± SD values of −197±0.79*‰* (*n* = 5) and −54.1±0.33*‰* (*n* = 5), respectively. All results are reported for non-exchangeable H expressed in the typical delta notation, in units of per mil (*‰*), and normalized on the Vienna Standard Mean Ocean Water – Standard Light Antarctic Precipitation (VSMOW-SLAP) standard scale.

For *δ* ^13^C and *δ* ^15^N analyses, between 0.5 and 1.0 mg of feather material was combusted online using a Eurovector 3000 (Milan, Italy - www.eurovector.it) elemental analyzer. The resulting CO_2_ and N_2_ was separated by Gas Chromatograph (GC) and introduced into a Nu Horizon (Nu Instruments, Wrexham, UK - www.nu-ins.com) triple-collector isotope-ratio mass-spectrometer via an open split and compared to a pure CO_2_ or N_2_ reference gas. Stable nitrogen (^15^N/^14^N) and carbon (^13^C/^12^C) isotope ratios were expressed in delta (*δ*) notation, as parts per thousand (*‰*) deviation from the primary standards: atmospheric nitrogen and VPDB (Vienna Pee Dee Belemnite carbonate) standards, respectively. Using previously calibrated internal laboratory C and N standards (powdered keratin and gelatin), within runs, precisions for *δ* ^15^N and *δ* ^13^C were better than ±0.15*‰*.

### Assigning origins

We tested for mean differences in feather *δ* ^2^H, *δ* ^13^C and *δ* ^15^N values between known breeding grounds (i.e. those grown locally) versus unknown wintering grounds using t-tests. Some individuals were sampled for both feathers grown on the breeding and wintering grounds. In those cases, we used a paired t-test to identify differences between growth origins. Multivariate Analysis of Variance (MANOVA) was used to test simultaneously for isotopic differences among feathers using Pillai's trace statistic. All statistical analyses were performed using R Version 2.10.1 [14].

Naturally molted tail feathers from 100 individuals from Argentina were used to assign Barn Swallows to their molting origins by using normal probability density functions [15]–[17] to assess the likelihood that the observed data (*δ* ^2^H_f_, *δ* ^13^C) could have resulted from growth at given locations within the *δ* ^2^H isoscape of [18] and *δ* ^13^C isoscape of [19] following methodology described in [15]. To this end, the amount-weighted mean precipitation *δ* ^2^H isoscape corresponding to the growing season of South America [18] was first converted into an equivalent feather *δ* ^2^H (*δ* ^2^H_f_) isoscape using the empirical equation reported for non ground-foraging Neotropical migrants [20]:
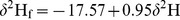
(1)


The expected standard deviation (σ_c_) among individuals growing their feathers at the same locality for *δ* ^2^H (σ = 14.4‰) was estimated using the standard deviation of the residuals from the same regression equation reported by [20]. To depict the probable molting origins of individuals, a normal probability density function (Equation 2) was applied to assess the likelihood that a given pixel in the *δ* ^2^H_f_ isoscape represented a potential origin for each feather sample:
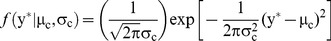
(2)where ƒ(y*|μ_c_,σ_c_) represents the probability that a given cell (pixel) represents a potential origin for an individual of unknown origin (y*), given the expected mean *δ* ^2^H_f_ for that cell (μ_c_) based on the predicted value for that cell within the isoscape, and the expected standard deviation (σ_c_) of *δ* ^2^H_f_ among individuals growing their feathers at the same locality.

A theoretical spatial *δ* ^13^C-distribution of vegetation in South America was obtained from [19], [21]. From this, we created a dichotomous surface of C3- and C4-dominated vegetation zones. Cells with *δ* ^13^C values<−20‰ were classified as C3-dominated and those with *δ* ^13^C values > −20‰ were classified as C4-dominated [22]. These zones were then converted to equivalent feather-*δ* ^13^C values assuming an isotopic discrimination factor of 2‰ between plant and feather, calculated based on known discrimination factors of ∼1‰ between plants and herbivorous insects [23]–[25], and ∼1‰ between insects and bird feathers [26]. The expected mean and standard deviation (SD) of feather-*δ* ^13^C for each region were calculated based on modeled *δ* ^13^C values extracted from the dichotomous feather-*δ* ^13^C isoscape. Equation 2 was then applied to assess the probability that the observed *δ* ^13^C of the feather represented growth in a C3 versus C4-dominated winter origin for each feather sample.

Following [15], our assignment algorithm used Baye's Theorem to compute the probability of each pixel *x_i_* being the origin of a feather sample, given the observed feather value *y_j_*, where *j* indexes the C3 or C4 vegetation zone.
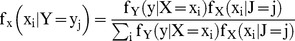
(3)


The random variables *Y* and *X* are both continuous and represent the observed feather-*δ* ^2^H values for Barn Swallows and the pixels within the feather-*δ* ^2^H isoscape, respectively. The random variable *J* is describes potential origins in C3 or C4-dominated zones and is thus categorical with a dimension of two.

Spatially explicit probability densities were normalized to the sum of likelihoods, thus yielding a single probability of origin surface for each feather sample. To statistically assign individuals to molt origin the calculated spatially explicit probability densities for each feather sample were reclassified using 3∶1 odds ratios of correctly assigning an individual to its molt origin. The set of cells that defined the upper 75% of estimated probabilities of origin was coded as “1” (likely) and the rest as “0” (unlikely) [17]. Results of the assignment of each individual were summed and mapped to obtain the most probable molting origin of the population. All analyses were performed using scripts adapted from [17] and employing the ‘raster’ package within R Statistical Computing environment, Version 2.10 [14] and ArcGIS Version 9.3 [27].

## Results

**Figure 1:**
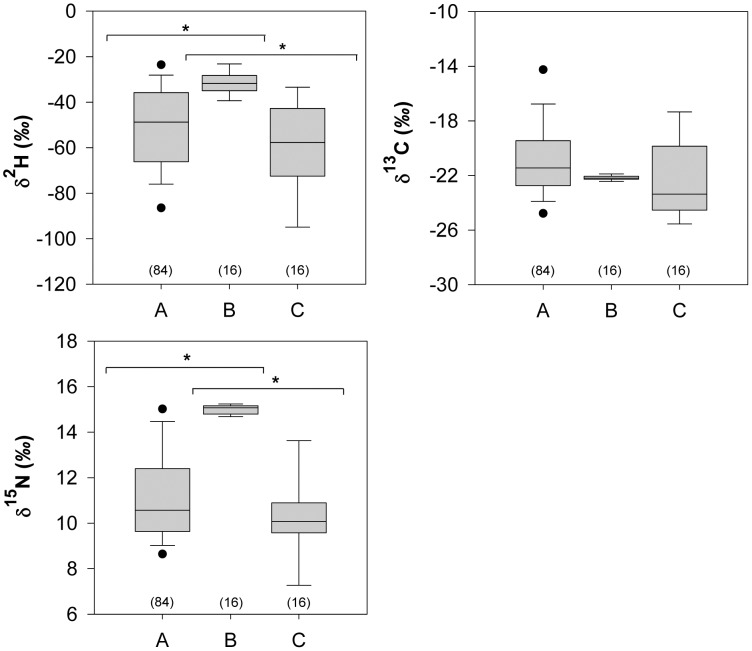
Boxplots of *δ* ^2^H, *δ* ^13^C and *δ* ^15^N values (‰) of Barn Swallow feathers. Letter A represents a sample of birds whose feathers were sampled once and were grown on unknown austral wintering grounds. Letter B and C represent feathers forced to grow on the breeding grounds in Argentina and those initially plucked (grown on unknown austral wintering grounds), respectively. Star symbol denotes significant differences in mean using independent (A vs C) or paired (B vs C) t-tests as appropriate (P<0.05). Numbers in brackets represent sample sizes. Results indicate that feathers grown on the breeding grounds were isotopically different from those grown on the austral wintering grounds for *δ* ^2^H and *δ* ^15^N values.

**Figure 2:**
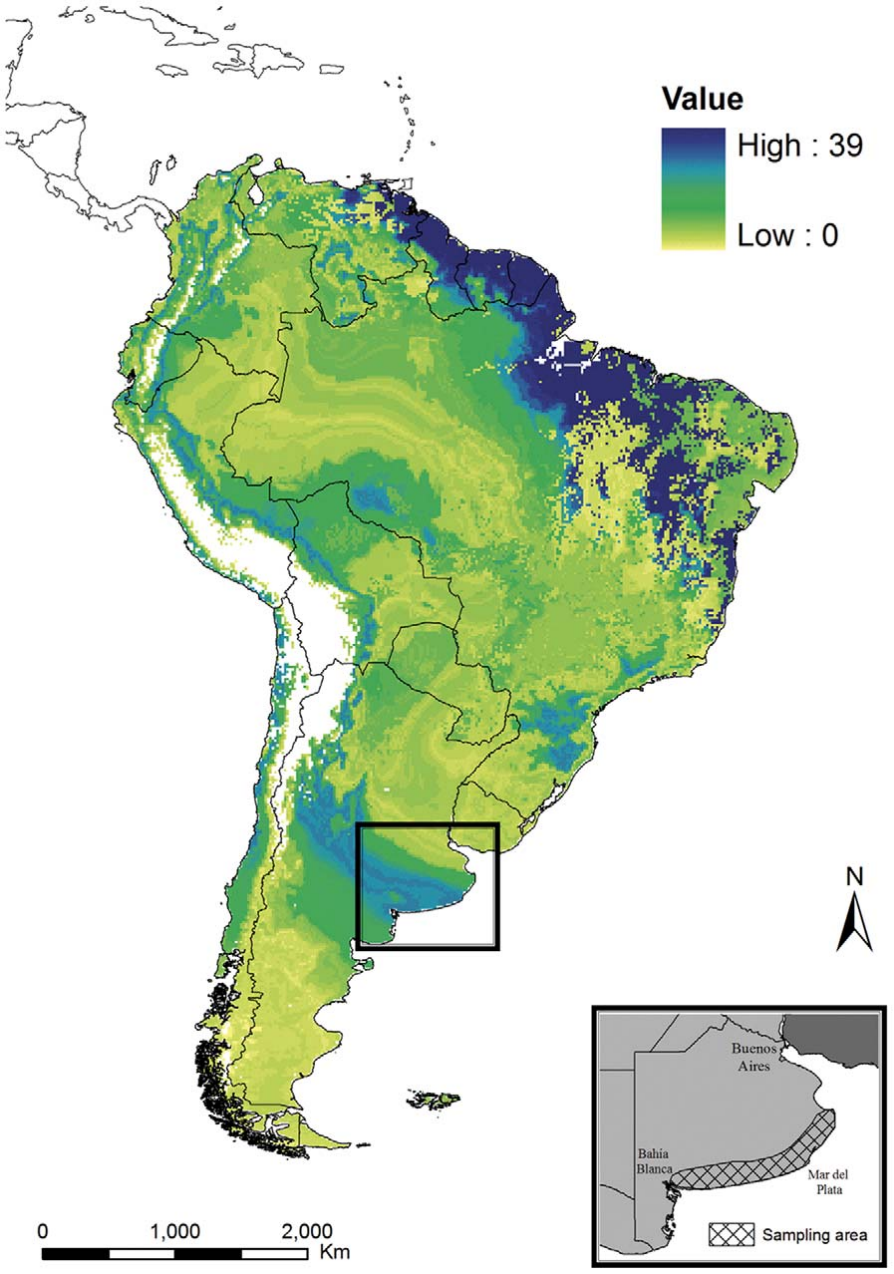
Potential molt origins of Barn Swallows breeding in the Atlantic coast of Buenos Aires province (Argentina). Maps were created using *δ* ^2^H and *δ* ^13^C values of winter-grown feathers. Values depicted on maps represent the number of individuals in the total sample that were assigned to each cell in the map, representing a potential molting origin according to a 3∶1 odds ratio.

Feathers grown on unknown wintering locations (*n* = 84) and those grown locally in Buenos Aires province (*n* = 16) differed significantly for the three isotopes simultaneously (MANOVA, *F*_3,96_ = 26.21, *P*<0.001) and for *δ* ^2^H (*t*_98_ = 3.96, *P*<0.001) and *δ* ^15^N (*t*_98_ = 8.06, *P*<0.001), but not in *δ* ^13^C values (*t*_98_ = −1.77, *P*>0.05) when tested separately (Figure 1). Similar results were found for feathers grown on unknown wintering (*n* = 16) and known breeding (*n* = 16) locations collected from the same individuals (Table 1). Feather types varied isotopically for the three isotopes (MANOVA, *F*_3,28_ = 32.06, *P*<0.001) as well as in *δ* ^2^H (t_15_ = 4.98, *P*<0.001) and *δ* ^15^N (t_15_ = 8.04, *P*<0.001) but not in *δ* ^13^C values (t_15_ = −0.17, *P*>0.1) (Figure 1). Feathers grown on the Argentinean breeding grounds (*n* = 16) formed a tight group (SD: *δ* ^15^N = 0.2*‰*, *δ* ^2^H = 5.0*‰*, *δ* ^13^C = 0.2*‰*), while feathers grown outside of the breeding season (*n* = 100) were more broadly distributed (SD: *δ* ^15^N = 2.0*‰*, *δ* ^2^H = 20.7*‰*, *δ* ^13^C = 2.8*‰*) (Figure 1). Molt origins based on *δ* ^2^H and *δ* ^13^C values corresponded primarily to regions in north-eastern South America, including north-eastern Brazil, French Guiana, Suriname, Guyana, and Venezuela (Figure 2).

**Table 1:** Mean and SD of *δ* ^2^H, *δ* ^13^C and *δ* ^15^N values (*‰*) in feathers of Barn Swallows captured on their breeding grounds in Buenos Aires province.

Growing location		n	mean	SD
***δ*** **^2^H**				
Wintering grounds	(A)	84	−52.3	20.7
Breeding grounds	(B)	16	−31.7	5.0
Wintering grounds	(C)	16	−57.2	20.6
***δ*** **^13^C**				
Wintering grounds	(A)	84	−20.9	2.8
Breeding grounds	(B)	16	−22.2	0.2
Wintering grounds	(C)	16	−22.0	3.2
***δ*** **^15^N**				
Wintering grounds	(A)	84	11.0	2.0
Breeding grounds	(B)	16	15.0	0.2
Wintering grounds	(C)	16	10.5	2.4

(A), feathers sampled once. (B), feathers forced to grow in known breeding grounds. (C), feathers initially plucked from birds sampled in (B).

## Discussion

Feathers induced to grow by swallows breeding in Buenos Aires province differed isotopically from those grown outside of the breeding season presumably during the austral winter. Thus, the ancestral (boreal) molt phenology was replaced by a new (austral) migratory strategy in this newly formed breeding population. Our isotopic assignment of wintering locations corresponded with the northeastern region of South America (specifically northern Brazil, French Guiana, Suriname, Guyana, and Venezuela, areas known to also be frequented by wintering swallows from North America [28], [29]. These findings agree with previous reports of sporadic observations of Barn Swallow in northern South America during the months of June, July and August [29]. Possibly, birds from this disjunct breeding population winter in areas that are available following the departure of North American migrants to their breeding grounds. This change of migratory strategy may have involved a complete change in molt strategy motivated by an adaptation to the new annual cycle particular to the southern hemisphere. The adoption of an austral migration and molt strategy by a founder population established in the opposite hemisphere clearly represents an extreme case of rapid adaptation which has been previously shown in a few species such as the Leach's Storm Petrel (*Oceanodroma leucorhoa*), a European long-distance migrant which has founded a new breeding population within its wintering range in South Africa [30].

The molt schedule for the new disjunct population of swallows within the annual cycle was the same as that for the ancestral population, namely following breeding and presumably on the austral wintering grounds. Thus, rather than adjusting their molt cycle to new environmental conditions, the Argentinean population presumably used the same cues as those used by the ancestral populations. Molt in birds is triggered by thyroid hormones in turn modulated by photoperiod [7], [8], [31]. A profitable future investigation would potentially be to monitor molt in captive Argentinean breeding birds to establish the calendar of molt directly.

The broad range of feather isotope values representing the austral wintering period of swallows breeding in Argentina suggest a number of possible molting areas in the southern portion of their breeding range. Indeed, we cannot rule out the possibility that some of the individuals we examined were in fact new arrivals from North America [12] or represented an extreme case of double breeding [32]. However, the most parsimonious explanation is the adoption of an austral migratory system that has Argentinean breeders wintering and molting over a broad range of northeastern South America. We recognize the ambiguity of assignment of birds to regions of South America using stable isotope methods. Indeed, little ground truthing of our multi-isotope feather isoscape for South America has been conducted and so ours represents only the most parsimonious of several possible explanations. Nonetheless, ours is a falsifiable hypothesis that can now be investigated further using isotopic tools and also the use of light-sensitive geolocators that are now small enough to be used on this species [33], [34].
